# Imitation by combination: preschool age children evidence summative imitation in a novel problem-solving task

**DOI:** 10.3389/fpsyg.2015.01410

**Published:** 2015-09-28

**Authors:** Francys Subiaul, Edward Krajkowski, Elizabeth E. Price, Alexander Etz

**Affiliations:** ^1^The George Washington University, WashingtonDC, USA; ^2^Centre for Behaviour and Evolution, Institute of Neuroscience, Newcastle UniversityNewcastle upon Tyne, UK

**Keywords:** imitation, social learning, innovation, cultural learning, problem-solving, cumulative culture, children, learning

## Abstract

Children are exceptional, even ‘super,’ imitators but comparatively poor independent problem-solvers or innovators. Yet, imitation and innovation are both necessary components of cumulative cultural evolution. Here, we explored the relationship between imitation and innovation by assessing children’s ability to generate a solution to a novel problem by imitating two different action sequences demonstrated by two different models, an example of imitation by combination, which we refer to as “summative imitation.” Children (*N* = 181) from 3 to 5 years of age and across three experiments were tested in a baseline condition or in one of six demonstration conditions, varying in the number of models and opening techniques demonstrated. Across experiments, more than 75% of children evidenced summative imitation, opening both compartments of the problem box and retrieving the reward hidden in each. Generally, learning different actions from two different models was as good (and in some cases, better) than learning from 1 model, but the underlying representations appear to be the same in both demonstration conditions. These results show that summative imitation not only facilitates imitation learning but can also result in new solutions to problems, an essential feature of innovation and cumulative culture.

## Introduction

Human children have been described as “cultural magnets” (Flynn, 2008), absorbing and transmitting the habits of their parents and society as a whole with exquisite fidelity. Yet, despite children’s exceptional imitative abilities as well as their sophisticated causal (Gopnik et al., 2001; Gopnik and Schulz, 2004) and technological (Defeyter et al., 2009; Cook and Sobel, 2011) knowledge, children are poor problem-solvers or innovators (Cutting et al., 2011; Beck et al., 2012; Chappell et al., 2013; Nielsen et al., 2014b). In a series of studies, Beck et al. (2011), Chappell et al. (2013) demonstrated that children younger than seven excel at imitating tool-making for the purposes of achieving a goal (i.e., tool-manufacture), but these same children cannot independently make the same tool to achieve the same goal (i.e., tool-innovation). This result is not restricted to urban children who might have few pressures to innovate given the availability of mass-produced toys. Cross-cultural research shows that San children in Southern Africa—where few commercial toys are available and there is considerable pressure to create new toys and recreational activities—are also poor problem-solvers or innovators (Nielsen et al., 2014b). Equally surprising is the fact that when tasks are made sufficiently complex, human adults are also poor innovators. In fact, novel innovations or independent invention is rare in adult humans (Lewis and Laland, 2012; McCaffrey, 2012). Together, these results indicate that while humans excel at imitating and propagating existing cultural practices (i.e., cultural transmission), they are poor at creating novel cultural variants, themselves.

Such results have led many to conceptualize imitation and innovation as mutually exclusive concepts (Ramsey et al., 2007; Legare and Nielsen, in press). According to this view, whereas imitation is a quintessential *social* learning mechanism involving the faithful reproduction of others’ responses, innovation is thought of as the prototypical *asocial* learning process that involves independently generating solutions to problems (Kummer and Goodall, 1985; Ramsey et al., 2007; Reader et al., 2011; Legare and Nielsen, in press). For instance, Ramsey et al. (2007) in a review of the literature describe innovation as, “…the process that generates in an individual a novel learned behavior that is not simply a consequence of social learning…” (p. 395). But what if problem-solving or innovation is not primarily the result of novel independent discovery, at which children and adults are generally poor, but is instead mediated in some instances by imitative learning, a skill at which humans of all ages excel. Richerson and Henrich (2012) suggest that “Learning mechanisms that… blend information from different models allow learners to effectively aggregate information across models and reduce transmission noise” (p. 42). From this it follows that one way to individually generate novel behaviors (i.e., innovation) is through the aggregation and combination of responses from multiple models (i.e., social learning). That is, the novel, “individually” generated solution to a problem is the result of summing up different behaviors that were socially learned from different models. As such, imitation by combination may represent a middle ground between social and asocial learning, with imitation mediating the transmission of information from multiple models and the individual producing a new action that is an amalgamation or the summation of socially learned responses, akin to “the Ratchet Effect” (Tomasello et al., 1993).

But despite young children’s impressive imitative abilities, it is unclear to what degree young children, who stand to benefit the most from cultural learning, are simply “cultural magnets,” faithfully replicating what they’ve observed in an effort to solve *familiar* problems (Flynn, 2008) or whether children are also “cultural innovators,” individually combining different responses learned from different models to solve *novel* problems. While the former does not provide much opportunity for innovation given that the child only replicates existing behaviors without alteration, the latter affords greater behavioral flexibility, allowing children to aggregate multiple responses^1^ and sources of knowledge in an effort to find optimal solutions to new problems, something that is essential for cumulative cultural evolution (i.e., ‘the ratchet effect’). To that end, the present study asked: *Can preschool age children solve novel problems by combining different responses from different models?* To answer this question we used a novel problem box to assess preschool age children’s ability to combine different types of responses demonstrated by 2 model to solve a novel problem (or innovate)^2^.

Previous research has shown that children benefit from observing multiple models (Bandura and Menlove, 1968; Schunk, 1987; Herrmann et al., 2013). For instance, Schunk (1987) showed that 10-years-old children paired with different peers who demonstrated how to solve a math problem (e.g., subtracting fractions) learn better than children exposed to a single model. Herrmann et al. (2013) demonstrated a comparable effect with preschool age children using an instrumental task. However, in all these studies, the different models demonstrated the *same* response or rule type (e.g., solving fractions), rather than different responses or components of an event sequence. As such, in these studies there was no opportunity to combine different types of responses across models to achieve a goal (or optimal outcome). Nonetheless, there is evidence from research on children’s causal reasoning that preschool age children and even infants can combine the effects of different objects across different events to generate accurate causal inferences. For instance, using the “blicket detector” task, Gopnik and colleagues (Gopnik et al., 2001; Sobel and Kirkham, 2007; Walker and Gopnik, 2014) presented participants with various conditions where one or two objects alone or in combination activated the blicket detector. Children as young as 18 months of age made the correct inference regarding whether one or two objects were required to activate the blicket detector, combining the different effects of individual objects to generate an accurate causal inference. Although outside the social domain, these results demonstrate that very young children are capable of generating novel solutions to problems (i.e., how to activate the blicket detector) by aggregating and combining different sources of causal information across different conditions and objects.

The combination of imitative responses to solve a novel problem and innovate, however, may present children with a unique suite of challenges. Imitating actions on objects is a multi-sensory and computationally complex problem that involves identifying the relevant actions and their respective goals, accurately sequencing those actions and mapping them to targets in distinct location(s) in space, while generating and executing a matching motor plan that may or may not be visually opaque (Nehaniv and Dautenhahn, 2002; Brass and Heyes, 2005). These challenges are compounded when the task requires imitatively combining different types of responses across different models separated by time and space. Specifically, keeping track of different individuals, copying different actions, while ignoring irrelevant information such as differences in size, posture or dress, should increase memory, attention and inhibitory demands. This is a particular concern given that preschoolers have poor executive functioning skills; specifically, poor inhibitory control and attention (Garon et al., 2008; Best and Miller, 2010), which are factors that are known to dampen imitation fidelity (Subiaul and Schilder, 2014).

In Experiment 1, we presented preschool age children with a problem box. We used a problem box because a number of studies have shown that preschoolers are exceptionally accurate at imitating multi-step responses using problem boxes (Horner and Whiten, 2005; Nielsen, 2006; Hopper et al., 2007; Lyons et al., 2007, 2011; McGuigan et al., 2007). Using this task we sought to answer the following questions: (a) Do children imitatively combine responses across models when problem-solving? Specifically, when problem-solving do children imitate both demonstrated responses relative to a Baseline condition, where no demonstration is provided? And, (b) When problem-solving, is imitation fidelity in the 2 model demonstration comparable to imitation fidelity in the 1 model demonstration where children do not have to imitatively combine responses?

*Hypotheses*: If children problem-solve by summative imitation, those in the 2 model demonstration condition should (a) generate more target responses than children in Baseline, (b) open both compartments more often than children in Baseline, and (c) performance should not significantly differ from children who learned from a single model.

## Experiment 1

### Methods

#### Participants

A total of 77 children (Females = 44), ranging in age from 3 to 5 years (*M* = 3.88, *SD* = 0.73) were recruited and tested in the Discovery Room in the National Museum of Natural History, Smithsonian Institute, Washington, DC, USA using approved IRB protocols from both the Smithsonian and the George Washington University. Eight other children were excluded due to video recording errors and four additional children were excluded due to experimenter error. We received informed consent from participants’ parent(s) or legal guardian(s), and we obtained informed assent from the child immediately prior to testing.

#### Materials

**FIGURE 1:**
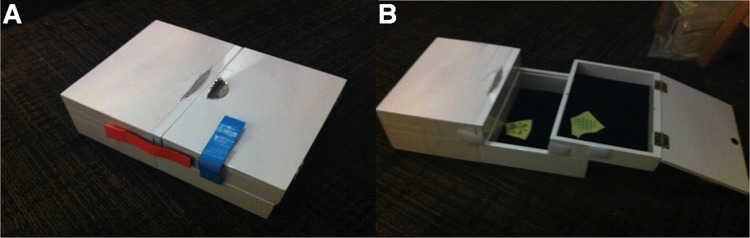
**Problem box task. (A)** Closed problem box showing the two defenses (blue and red). **(B)** Opened problem box showing both upper and lower compartments.

The experimental apparatus was a problem box with two compartments (upper, lower) and two “defenses” consisting of Velcro strips (top, side) in distinct colors (red, blue) that prevented the compartments from opening (**Figure 1**). Two stickers were hidden in each compartment. After the child found the stickers, they placed them on a white piece of paper (8.5 in. X 11 in.). The experiment was video recorded for data coding at a later time. In order to simplify the task, only half of the box was rendered operable.

#### Experimental Groups

Groups included a trial and error (Baseline) learning group and two experimental demonstration (1 and 2 model) conditions in which children first observed a model(s) demonstrate in person (live) how to open the box three consecutive times.

##### Baseline

An experimenter asked the child how many stickers they thought were in the box. Regardless of their answer, the experimenter said, “There are two stickers in the box.” And then, encouraged the child to find the two stickers in the box. No additional instruction or demonstration was provided.

##### Demonstration conditions

There were two types of demonstrations:

###### 1 Model demonstration

A model approached the box, said, “Watch me,” then removed the first defense (R) and opened (O) the corresponding compartment. The same model then proceeded to remove the second defense (R) and open the second (O) compartment (RORO). Then the model returned the box to its original state and repeated the actions described above two more times (three demonstrations opening the upper compartment and three demonstrations opening the lower compartment).

###### 2 Model demonstration

The first model approached the box, said, “Watch me,” removed the first defense (R) and then opened (O) the corresponding compartment. The same model then returned the box to its original state and repeated the demonstration two more times (three demonstrations opening one of the two compartments). Following the third demonstration, the model walked out of view of the child. A second model approached the box, said, “Watch me,” removed the second defense (R), and opened (O) the corresponding compartment (RO – RO). The second model then returned the box to its original state and repeated the demonstrated action two more times (three demonstrations opening the other compartment). Following the third demonstration, the model walked out of view of the child.

A third experimenter, who sat with the child throughout the demonstration, faced them and asked, “Do you remember how many stickers are in the box?” If the child answered correctly, the experimenter said, “That’s right! There are two stickers in the box. Can you find the two stickers in the box?” If they answered incorrectly, the experimenter said, “There are two stickers in the box. Can you find the two stickers in the box?”

Both demonstration conditions followed an alternating pattern, RO RO, where actions (defense removal) and goals (opening compartments) were presented in a causally logical, alternating fashion. Following each demonstration, the model returned the box to its original state and repeated this demonstration two more times. The number of demonstrations in the 1 and 2 model conditions was the same. In both demonstration conditions children saw the model(s) remove the Velcro strip and the corresponding compartment three times for each compartment. In all demonstrations, the order of opening each compartment was counterbalanced. In the 2 model demonstration, models were the same sex and the compartments they opened were counterbalanced between children. Conditions are summarized in **Table 1**.

**Table 1:** Summary of learning conditions.

Learning condition	Experiment 1: demonstration type: RO-RO	Experiment 2: demonstration type: RR-OO	Experiment 3: demonstration type: OO-RR
Baseline	No demonstration was provided	No demonstration was provided	No demonstration was provided
1 Model	Model 1: removes first defense then opens corresponding compartment (R, O). Model 1: removes second defense then opens corresponding compartment (R, O). Repeats two more times	Model 1: removes both defenses (R, R). Repeats two more times. A white barrier obscures the child’s view of the box (∼5 s). The box is prepared for the second demonstration. Model 1: opens both compartments (O, O). Repeats two more times	Model 1: opens both compartments (O, O). Repeats two more times. A white barrier obscures the child’s view of the box (∼5 s). The box is prepared for the second demonstration. Model 1: removes both defenses (R, R). Repeats two more times
2 Models	Model 1: removes first defense then opens corresponding compartment (R, O). Repeats two more times. Model 2: removes second defense then opens corresponding compartment (R, O). Repeats two more times	Model 1: removes both defenses (R, R). Repeats two more times. A white barrier obscures the child’s view of the box (∼5 s). The box is prepared for the second demonstration. Model 2: opens both compartments (O, O). Repeats two more times	Model 1: opens both compartments (O, O). Repeats two more times. A white barrier obscures the child’s view of the box (∼5 s). The box is prepared for the second demonstration. Model 2: removes both defenses (R, R). Repeats two more times

*Videos of each of the demonstration conditions can be seen here: https://www.youtube.com/watch?v=Zu3CNXoIaOs&index=1&list=PLft-Ni1aB5CWD_NRHotwvc5Mid0pRNKx-). Table summarizes the differences between the learning conditions.*

#### Measures

Trained coders analyzed the following responses and measures:

##### Target responses

There are a total of four target actions: (a) remove top Velcro defense, (b) remove side Velcro defense, (c) lift using top handle, (d) slide using top/side handle (c.f., **Figure 1**). The execution of each target response was coded as +1.

##### Errors

We code four types of errors: (a) trying to lift without removing the top defense, (b) trying to slide without removing the side defense, (c) trying to open the opposite side of the box, which was not operable, and (d) breaking apart the box by inappropriately opening a compartment (e.g., lifting the entire top compartment). Each error was coded as -1.

##### Fidelity score

This was a composite score that included the total number of target responses (+0–4) plus points for executing the individual target actions in the exact same order demonstrated by the model, including matching the demonstrated order of removing defenses (+0–1) and lifting/sliding actions (+0–1), minus the total number of errors (-0–4). Total fidelity score range: -4 to 6. This composite score measured how well individuals’ responses matched those demonstrated by the model(s) while excluding individual trial-and-error learning (e.g., by subtracting errors) or the use of idiosyncratic means to achieve the same result—emulation learning—(by evaluating order of target responses). Fidelity scores could only be generated for the demonstration conditions because the Baseline condition included no demonstration prior to testing as such there was no way to assess whether responses matched those of the model or not.

##### Opening Style

To further disambiguate imitation from emulation and establish a baseline rate of spontaneously opening the box using a particular method, we also evaluated whether children adopted a particular opening style. Specifically, there were two types of opening styles we evaluated, an alternating style (RO-RO) and a blocked style (RR-OO). Children in the demonstration conditions were given a score of 1 if they matched the opening style used by the model and a score of 0 if they did not.

##### Opened Both Compartments

This was a binomial measure that assessesd whether children opened both the upper and lower compartment of the box at least one time. If children opened both compartment one or more times they were given a score of 1. If they opened only 1 or neither compartment they were given a score of 0.

Two of the studies authors (AE, EK) and a third independent coder not involved with data collection or familiar with the study’s aims coded all responses (Experiments 1 and 2: AE; Experiment 3: EK). Inter-rater agreement (between AK or EK and a third independent coder) was high, κ = 0.75–0.98, across measures and studies (Experiments 1–3).

#### Statistical Analysis

We used non-parametric statistics when assessing binary or discontinuous measures such as the opening style score, opening both compartments and error type (Experiment 3). Parametric analyses were used for all other measures unless otherwise specified.

### Results

#### Was Learning in the Demonstration Conditions Better than Baseline?

Preliminary analyses showed no reliable indication of age or gender effects, so these factors were not analyzed further. A Univariate analysis of variance (ANOVA) comparing target responses across conditions (Baseline, 1 model, 2 model) was statistically significant [*F*(2,74) = 19.59, *p* < 0.001, η^2^ = 0.35]. Pairwise comparisons showed that children in both demonstration conditions made significantly more target responses (*M*_1_ = 3.82, 95% CI [3.48, 4.15], *M*_2_ = 3.93 [3.65, 4.22]) than children in Baseline (*M*_B_ = 2.68 [2.36, 2.99], *p*s < 0.001, *d*_1-B_ = 1.14 [0.57, 1.70], *d*_2-B_ = 1.25 [0.73, 1.78]). The difference between the demonstration conditions (*d*_2-1_ = 0.12 [-0.43, 0.66], *p* = 1.0) was not statistically significant.

We also compared the number of errors made by children in the different learning conditions. Results showed that there was a main effect for learning condition [*F*(2,74) = 19.26, *p* < 0.001, η^2^ = 0.34]. Pairwise comparisons revealed that children in the demonstration conditions (*M*_1_ = 0.27, 95% CI [0.05, 0.49], *M*_2_ = 0.07, 95% CI [-0.12, 0.26]) made significantly fewer errors than children in Baseline (*M*_B_ = 0.92, 95% CI [0.71, 1.13], *p*s < 0.001, *d*_1-B_ = 0.65 [-1.02, -0.27], *d*_2-B_ = -0.85 [-1.20, -0.51]). The differences between the demonstration conditions were not statistically significant (*d*_1-2_ = 0.21, 95% CI [-0.15, 0.56], *p* = 0.49, all tests are Bonferroni adjusted). Results are summarized in **Table 2**.

**Table 2:** Mean (SD) for the various measures used to evaluate performance.

Experiment: demonstration	Model condition	Target responses	Opened both compartments	Errors	Fidelity
Experiment 1: None	Baseline	2.68 (1.28)	0.32	0.92 (0.70)	N/A
Experiment 1: RO-RO	1 Model	3.82 (0.50)^∗^	0.86^∗^	0.27 (0.55)^∗^	3.62 (1.13)
Experiment 1: RO-RO	2 Model	3.93 (0.25)^∗^	0.93^∗^	0.07 (0.25)^∗^	4.43 (0.86)
Experiment 2: RR-OO	1 Model	3.33 (1.27)	0.74^∗^	0.92 (1.11)	2.44 (2.81)
Experiment 2: RR-OO	2 Model	3.61 (0.96)^∗^	0.78^∗^	0.75 (0.93)	2.93 (2.23)
Experiment 3: OO-RR	1 Model	3.28 (0.39)	0.56	1.16 (0.46)	N/A
Experiment 4: OO-RR	2 Model	3.48 (0.34)	0.78^∗^	1.57 (0.49)^∗^	N/A

*Demonstrations included two types of actions, remove defense (R) and open compartment (O). How these different actions were demonstrated was manipulated in each Experiment.*

*^∗^Significantly different when compared to Baseline, *p* < 0.05.*

Given that children in the demonstration conditions clearly evidenced social learning by virtue of generating more target responses than children in Baseline, we did not analyze Baseline performance further.

#### Was there Evidence of Imitation by Combination or Summative Imitation?

93% (28/30) of children in the 2 model condition opened both compartments, retrieving both stickers. This rate of response differed significantly from the Baseline rate (*M* = 0.32, *Z* = -4.72, *p* < 0.001, effect size *r* = 0.53, Mann–Whitney test). Among children in the 2 model condition who opened both compartments, 96% (27/28) used the demonstrated—alternating—method, where children removed a defense and then opened the corresponding compartment (RO-RO). Again, these rates differed from the Baseline rate of spontaneously using the RO-RO method (*Z* = -2.95, *p* < 0.01, *r* = 34, Mann–Whitney test).

#### Did Imitation Fidelity Differ Between the 1 and 2 Model Demonstration Conditions?

**FIGURE 2:**
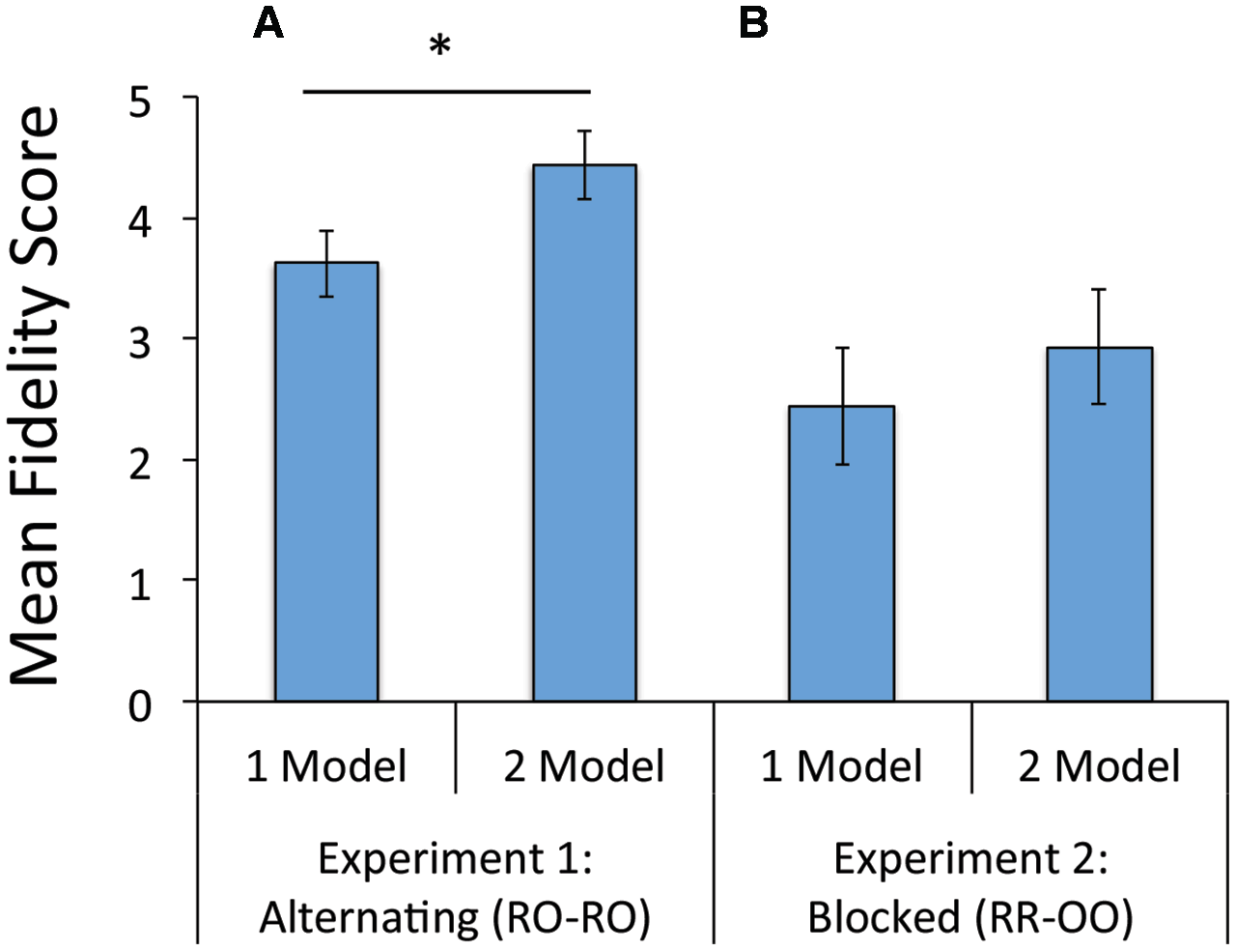
**Mean imitation fidelity score in the 1 and 2 model demonstrations conditions: **(A)** Experiment 1 and **(B)** Experiment 2.** ^∗^*p* < 0.05.

Fidelity scores were higher in the 2 model condition (*M*_2_ = 4.43 [4.41, 4.80]) than the 1 model condition (*M*_1_ = 3.68 [3.26, 4.11]), and this difference (*M*_2-1_ = -0.75 [-1.31, -0.19]) reached significance [*F*(2,49) = 7.31, *p* = 0.009, η^2^ = 0.13, Univariate ANOVA). Results are summarized in **Figure 2A**.

### Discussion

Results show that children successfully imitate different events demonstrated by different models, solving a novel problem by summative imitation. Specifically, children in the 2 model demonstration condition generated more target responses and opened both compartments more often than children in Baseline. Unexpectedly, children in the 2 model condition imitated with greater fidelity when compared to children in the 1 model condition. This difference is best explained by the fact that children in the 2 model condition made (marginally) fewer errors. These results confirm that children are not only adept at imitating with high-fidelity the responses of a single model but that they can imitate with high-fidelity across multiple models and effectively sum up different modeled actions or events to achieve a novel goal.

However, because models demonstrated an alternating technique where compartments were opened immediately after the removal of a defense, it is possible that children may not have imitated but rather learned about the causal affordances associated with opening the box. That is, each defense had to be removed in order to open each compartment. To test this alternative explanation for the results of Experiment 1, Experiment 2 evaluated whether children evidence summative imitation when the actions (i.e., defense removal or R) and the goals (opening compartment or O) are temporally and causally disconnected and demonstrated by different models (e.g., RR-OO). If children are learning about the causal affordances of the task, rather than imitating by combining the model’s responses, then they should open the box using the alternating technique (i.e., RO-RO) as opposed to the demonstrated method (RR-OO). To that end, Experiment 2 sought to replicate the results of Experiment 1 and, additionally, address whether children can learn by summative imitation in a more causally opaque task where 1 model removes both defenses and another opens both compartments.

*Hypotheses*: Same as in Experiment 1.

## Experiment 2

### Methods

#### Participants

An additional 55 children (Females = 28) ranging in age from 3 to 5 years (*M* = 3.98, *SD* = 0.80) were recruited and tested using the same procedures described above for Experiment 1. Two children were excluded due to experimenter error.

#### Task

Same as in Experiment 1.

#### Procedures

All procedures were identical to those of Experiment 1 except that a large white poster board was used to conceal the box before, between, and after demonstrations to obscure additional manipulations to prepare the box—limiting access to causal information. Children were tested in one of the following social learning conditions.

##### Baseline

Because this was a trial and error learning condition, we did not re-collect Baseline data for Experiment 2. As such, we compared performance in Experiment 2 with Baseline performance collected for Experiment 1.

###### 1 Model Demonstration

A model approached the box, said “Watch me,” removed both defenses (RR) then returned the box to its original state. This procedure was repeated two more times (three demonstrations removing defenses). Following the third demonstration, a white barrier obscured the child’s view of the box (∼3–5 s) during which time the box was prepared for the second demonstration. Once the box was reconfigured, the same model said “Watch me,” then opened both compartments (OO). Once the model opened each compartment, the model closed both compartments. This procedure was repeated two more times (three opening both compartments).

###### 2 Model Demonstration

One model approached the box, said “Watch me,” removed both defenses (RR) in succession and then returned the box to its original state, repeating two more times (three demonstrations removing defenses). Following the third demonstration, a third experimenter obscured the child’s view of the box (∼5 s) with a white barrier during which time the box was prepared for the second demonstration by a different model. Specifically, the defenses were removed and placed in front of the box. Before the barrier was raised again, the first model walked out of view of the child. At this point, the barrier was raised (by a third experimenter), a second model approached the box, said “Watch me” then demonstrated opening each compartment in succession (OO). Following each demonstration, the model closed both compartments. This procedure was repeated two more times (three demonstrations opening compartments). Following the third demonstration, the model walked out of view of the child. All other aspects of the procedures were identical to those described above for Experiment 1.

Following both demonstration conditions (1 or 2 models), the third experimenter then asked children the number of stickers in the box. Regardless of their answer, the third experimenter encouraged the child to find the two stickers in the box using the same procedures described for Experiment 1. See **Table 1** for differences between learning conditions across Experiments.

In both 1 and 2 model demonstration conditions children saw an equal number of demonstrations removing defenses and opening compartments. In both demonstration types, the resulting demonstration followed a blocked pattern, RR – OO, where actions (defense removal) and goals (opening compartments) were presented separately. In all demonstrations, the order of opening each compartment was counterbalanced. In the 2 model demonstration, models were the same sex and, as in the 1 model demonstration condition, the compartments they opened were counterbalanced between children.

#### Coding, Measures, and Hypotheses

Same as Experiment 1.

### Results

#### Was Learning in the Demonstration Conditions Better than Baseline?

Preliminary analysis showed a reliable indication of age effects but not gender effects, so age was included as a covariate in subsequent analyses. A Univariate ANOVA comparing the number of target responses across groups (Baseline, 1 model, 2 model) and including age as a covariate was significant [*F*(2,79) = 3.838, *p* = 0.03, η^2^ = 0.09]. Corrected for age, the demonstration conditions showed a linear pattern, with performance in the 2 model condition being the highest (*M*_2_ = 3.57 [3.14, 4.01]), followed by 1 Model (*M*_1_ = 3.34 [2.91, 3.79]) and, finally, Baseline (*M*_B_ = 2.78 [2.26, 3.28]). Pairwise comparisons showed that only the 2 model condition was reliably better than Baseline (*M*_2-B_ = 0.85 [0.78, 1.61], *p* = 0.03; *M*_1-B_ = 0.63 [-0.15, 1.39, *p* = 0.16], Bonferroni adjusted). The performance in the 2 model condition was not reliably better than performance in the 1 Model condition, however (*M*_2-1_ = 0.23 [-0.52, 0.98], *p* = 0.31, Bonferroni adjusted). As in Experiment 1 we compared the number of errors made by children in the different learning conditions. Results showed that there was no main effect for learning condition [*F*(2,74) = 2.73, *p* = 0.73, η^2^ = 0.01]. Results are summarized in **Table 2**.

#### Was there Evidence of Imitation by Combination or Summative Imitation?

79% (22/28) of children in the 2 model condition opened both compartments, retrieving both stickers. This rate of response significantly differed from Baseline rates (*M* = 0.32, *Z* = -3.52, *p* < 0.001, *r* = 0.50, Mann–Whitney test). Of the children in the 2 model condition who opened both compartments, 90% (20/22) used the demonstrated—blocked—method (RR-OO). Again, these rates differed from Baseline rates of spontaneously using the RR-OO method (*Z* = -6.14, *p* < 0.001, *r* = 0.87, Mann–Whitney test). Results are summarized in **Table 2**.

#### Did Imitation Fidelity Differ between the 1 and the 2 Model Demonstration Conditions?

Preliminary analyses revealed that imitation fidelity did not differ by age so age was excluded from further analysis. While imitation fidelity was greater in the 2 model (*M* = 4.43 [3.38, 5.47]) than in the 1 model demonstration condition (*M* = 3.85 [2.78, 4.92]), this difference was not statistically significant [*F*(1,54) = 0.559, *p* = 0.44, η^2^ = 0.01, Univariate ANOVA]. Results are summarized in **Figure 2B**.

### Discussion

Results from Experiment 2 largely replicate those reported for Experiment 1 using a more challenging procedure than the one used in Experiment 1 where actions and goals were presented separately. This feature of the demonstration made the causal link between removing the defenses before opening a compartment ambiguous. As such, it should not be surprising that children generally performed worse across demonstration groups in comparison to children in Experiment 1. This result is consistent with work by Bauer (1992) and Bauer and Hertsgaard (1993) showing that in an elicited imitation task, young children recall events that are causally linked more effectively than event sequences that are arbitrarily associated. In contrast to the results of Experiment 1, children’s fidelity scores in the 2 model condition was not significantly better than those of children in the 1 model condition. One reason for this might have to do with the introduction of the barrier in between demonstrations which might have added to children’s cognitive load. Nonetheless, as in Experiment 1, children in the 2 model condition not only generated significantly more target responses and opened both compartments more often than children in Baseline, their imitation fidelity did not significantly differ from that of children in the 1 model demonstration condition. This result is consistent with the hypothesis that summative imitation—imitatively combining different actions demonstrated by two or more models—is equivalent to imitative learning from a single model (where no combination is required).

The fact that children in the 2 model condition adopted the style demonstrated (i.e., RR-OO) rather than an alternative method (e.g., RO-RO), shows that children were imitating the demonstrated technique rather than achieving the same goal via affordance learning, end-state emulation or goal emulation (Whiten, 2008; Whiten et al., 2009). Children in Experiment 2, however, performed slightly worse than those in Experiment 1. This difference may be explained by the fact that children in Experiment 2 generally paused after opening each compartment to remove the sticker (increasing trial duration). Pausing to retrieve stickers likely increased the likelihood of forgetting which target actions had already been achieved, resulting in the repetition of already completed target responses or the execution of irrelevant responses such as closing opened compartments after the sticker had been removed. Other researchers have reported similar response patterns (e.g., Horner and Whiten, 2005).

Nonetheless, Experiments 1 and 2 makes clear that children imitate each event demonstrated with great fidelity, regardless of whether those events are demonstrated by 1 or 2 models. However, it is less clear whether children in the 1 and 2 model condition encode the two different action events (RR, OO) the same way. Specifically, whether children in the 1 and 2 model demonstration condition encode events flexibly, whereby, for example, RR and OO can be recalled in different orders (i.e., RR – OO or OO – RR) or whether they are encoded and subsequently recalled in the demonstrated order. While learning may generally be comparable between 1 and 2 models, there might be differences in how flexibly children learn the sequence of events in each demonstration condition. The work on overimitation suggests that when interacting with artifacts children are remarkably inflexible, imitating with high-fidelity even when some of the action are causally meaningless and costly (Lyons et al., 2007, 2011; Lyons, 2009). But, there is also evidence that children imitate flexibly and selectively, taking into consideration various social variables including the social context (Nielsen et al., 2012), task-difficulty (Williamson and Meltzoff, 2011), physical constraints (Gergely et al., 2002) and model’s intent (Lyons et al., 2011) to name a few (for a review see: Over and Carpenter, 2012).

The relatively lower imitation fidelity of children in the 1 model condition might suggest that children in that condition are more flexible and may imitate more selectively than children in the 2 model demonstration condition. Perhaps the causal affordances in the 1 model condition were more salient than the model’s actions, leading children to focus on the affordances of the task and less on specific actions. Alternatively, children in the 2 model condition may have done better, in general, not because they imitated each model’s actions faithfully but because, in the course of faithfully imitating each model’s actions, they learned the causal constraints of the task better than children in the 1 model condition.

Having established that children can accurately combine two different demonstrated events across different models in Experiments 1 and 2, Experiment 3 sought to assess the flexibility of children’s ability to imitatively combined different responses in the course of solving a novel problem by summative imitation. To do this, Experiment 3 replicated the methods used in Experiment 2 but reversed the order of the events demonstrated: Children first observed compartments being opened prior to the defenses being removed, violating causality.

## Experiment 3

### Methods

#### Participants

A total of 49 children (Females = 23), ranging in age from 3 to 5 years (*M* = 3.88, *SD* = 0.73) were recruited from the Discovery Room in the National Museum of Natural History, Smithsonian Institute, Washington, DC, USA.

One other child was tested but excluded due to experimenter error. We received informed consent from participants’ parent(s) or legal guardian(s), and we obtained informed assent from the child immediately prior to testing.

#### Materials

Same as Experiments 1 and 2.

#### Experimental Groups

Same as in Experiment 2 with the following exception:

##### 1 Model Demonstration

A model approached the box, said “Watch me,” opened the first compartment (O) and then proceeded to open the second (O) compartment (O, O). This was repeated two additional times (three demonstrations opening each compartment). After the third demonstration, a third experimenter, briefly, blocked the child’s view of the box with a white barrier (∼5 s). During this time, the Velcro defenses were added. Once defenses were in place, the barrier was removed and the same model said, “Watch me” then proceeded to remove each defense (R, R) in sequence three consecutive times (three demonstrations removing defenses).

##### 2 Model Demonstration

The first model approached the box, said “Watch me,” opened the first compartment (O) and then proceeded to open the second (O) compartment (O, O). The same model then returned the box to the starting state and repeated the demonstration two more times (three demonstrations opening each compartments). After the third demonstration, a third experimenter, briefly, blocked the child’s view of the box with a white barrier (∼5 s). During this time, the Velcro defenses were added. Once defenses were in place, the first model walked out of view of the child, a third experimenter removed the barrier, a second model approached the box, said “Watch me” and demonstrated removing each defense in sequence (R, R). The same model then returned the box to the starting state and repeated the removal of defenses two more times (three demonstrations removing defenses). Following the third demonstration the second model walked out of view of the child.

As in Experiments 1 and 2, following both demonstration conditions (1 or 2 models), the third experimenter then asked children the number of stickers in the box. Regardless of their answer, the experimenter encouraged the child to find the two stickers in the box.

As in the previous experiments, the number of demonstrations in the 1 and 2 model conditions was the same. In all demonstrations, the order of opening each compartment was counterbalanced as was the removal of defenses. All other procedures were identical to those described for Experiment 1. Please refer to **Table 1** for a summary of the procedures in the different learning conditions across Experiments.

Note that in contrast to Experiments 1 and 2, if children imitate the model faithfully (by attempting to open the compartments before removing the defenses) they will make lift and/or slide error(s). Counter-intuitively, in Experiment 3, more errors, specifically, more lift and/or slide errors, corresponds with more faithful imitation.

#### Measures

Same as Experiments 1 and 2.

#### Statistical Analysis

Same as above.

### Results

#### Did Children in the Demonstration Condition Make More Target Responses than Children in Baseline?

Preliminary analyses revealed that age significantly correlated with target responses (*r* = 0.33, *p* < 0.01, Pearson correlation) as such we included age as a covariate. A Univariate ANOVA with number of target responses as the dependent measure, number of models as a fixed factor and age as a covariate produced a main effect for age [*F*(2,72) = 6.81, *p* = 0.01, η^2^ = 0.90] and a marginally significant effect for number of models [*F*(2,72) = 2.50, *p* = 0.09, η^2^ = 0.70]. However, pairwise comparisons using the Bonferroni correction procedure revealed no significant differences between conditions, Baseline vs. 1 vs. 2 models (all *p*s > 0.10). Results are summarized in **Table 2**.

#### Did Children in the Demonstration Conditions Successfully Open Both (Upper and Lower) Compartments More Often than Children in Baseline?

As was done above in Experiments 1 and 2, Mann–Whitney tests were used to compare 1 and 2 model demonstration conditions to Baseline. When compared to children in Baseline (*M*_B_ = 0.32), significantly more children in the 2 model (*M*_2_ = 0.78) but not in the 1 model (*M*_1_ = 0.56) demonstration condition opened both compartments (*M*_1_: *Z* = -1.69, *p* = 0.18, *r =* 0.20; *M*_2_: *Z* = -3.07, *r* = 0.36, *p* < 0.01, *p*-values are corrected for multiple comparisons using the Bonferroni procedure). In contrast to Experiments 1 and 2, the high frequency of errors made by children in Experiment 3 made it difficult to accurately estimate fidelity scores as was done in the previous studies. As such, these analyses are omitted here.

#### Were there Differences in the Total Number of Errors Children Made Across the Different Conditions?

Preliminary analysis revealed that age did not significantly correlate with the number of errors children made (*r* < 0.20, *p* > 0.10), as such we did not analyze age further. A Univariate ANOVA comparing the number of errors across learning conditions was marginally significant [*F*(2,72) = 3.01, *p* = 0.06, η^2^ = 0.08). Children in the 2 model demonstration condition made the most errors (*M*_2_ = 1.57 [1.18, 1.95], *M*_1_ = 1.16 [0.79, 1.53], *M*_B_ = 0.92 [0.55, 1.29]). Pairwise comparisons showed that children in the 2 Model condition made marginally more errors than children in Baseline (*M*_2-B_ = 0.65 [-0.88, 0.40], *p* = 0.052; *M*_1-B_ = 0.24 [-0.40, 0.88, *p* = 1.00], Bonferroni adjusted). However, children in the 2 Model condition did not reliably make more errors than children in the 1 model condition (*M*_2-1_ = 0.41 [-0.25, 1.10], *p* = 0.39, Bonferroni adjusted). Results are summarized in **Table 2**.

**FIGURE 3:**
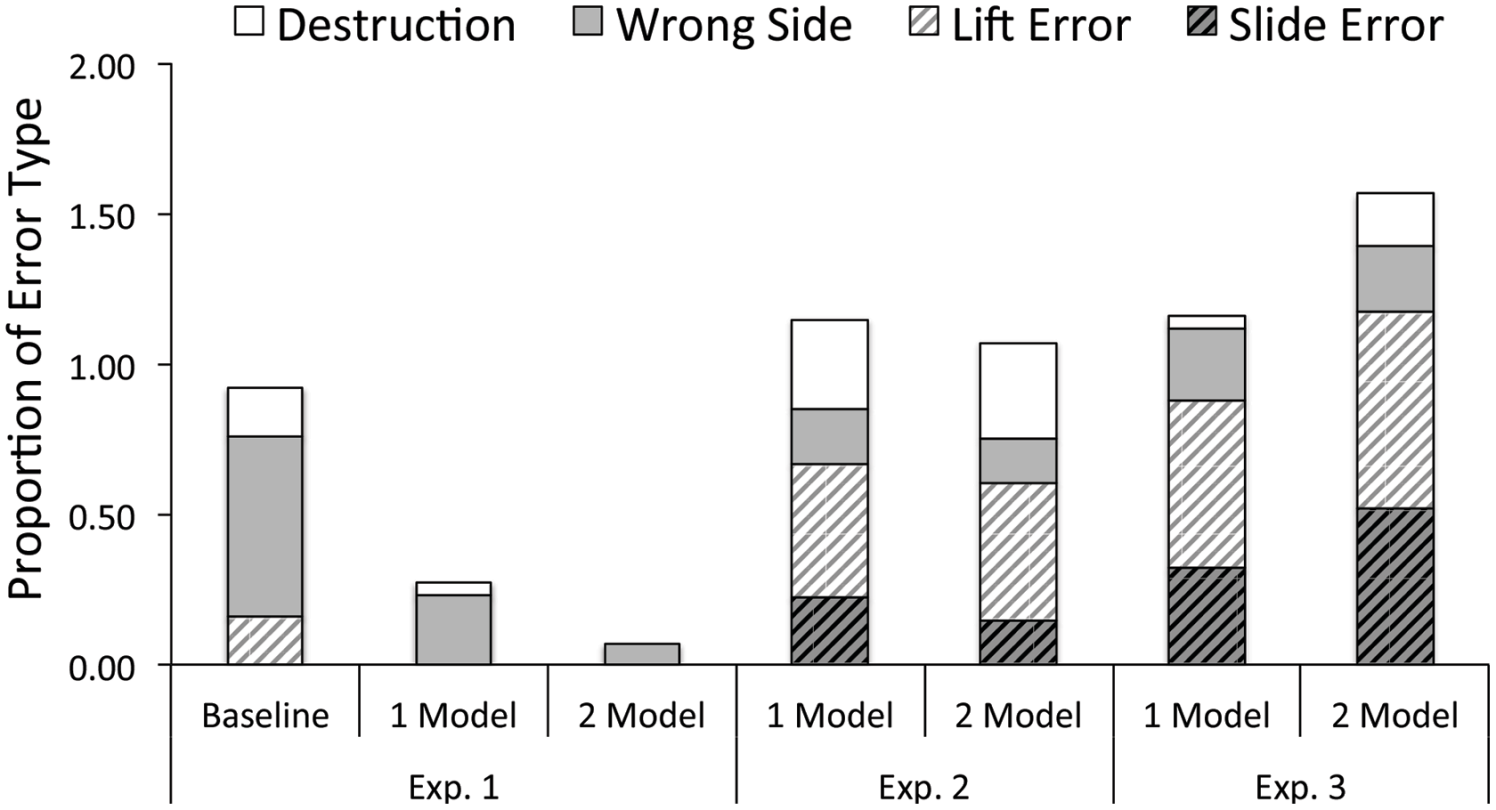
**Summary of error types by condition and experiment**.

To disambiguate random errors from imitation-related errors, we included an analysis of errors based on learning condition (i.e., Baseline, 1 Model, 2 Model). Specifically, we analyzed whether there were differences in the types of errors children made across learning conditions. Children in the 1 and 2 models demonstration conditions did not make different types of errors (all *Z*s < 1.50, *p*s > 0.10, *r*s < 0.18, Mann–Whitney test). However, compared to Baseline, children in both demonstration conditions made significantly more demonstration-related errors (slide: *Z* = -3.05, *p* < 0.03, *r* = 0.43, lift errors: *Z* = 2.92, *p* < 0.03, *r* = 0.41) as well as one non-demonstration related error such as interacting with the wrong side of the box (wrong side: *Z* = -2.55, *p* = 0.03, *r* = 0.36). Learning conditions did not differ in terms of breaking the box while trying to find the stickers (destroy: *Z* = -1.40, *p* = 0.48, *r* = 0.20). All analyses have been corrected for multiple comparisons using Bonferroni Procedure. Results are summarized in **Figure 3**.

#### Did Children in the Demonstration Conditions of Experiment 3 Make More Errors than Children in the Demonstration Conditions of Experiments 1 and 2?

To answer this question we performed a Univariate ANOVA that included number of errors as the dependent measure and experiment (1–3) and number of models (0, 1, 2) as fixed factors. Results showed a main effect for Experiment, *F*(2,229) = 17.92, *p* < 0.001, η^2^ = 0.14, but not for number of models [*F*(2,229) = 0.64, *p* = 0.53, η^2^ = 0.01]. There was also a significant interaction between number of models and Experiment, *F*(4,229) = 5.67, *p* < 0.001, η^2^ = 0.09. To understand the number of models by Experiment interaction, recall that in Experiment 1 children in both demonstration conditions (*M*_1_ and *M*_2_) made significantly fewer errors than children in Baseline. Whereas, in Experiment 3, children in the 2 Model (but not 1 model demonstration) condition made marginally more errors than children in Baseline. In Experiment 2, children in the demonstration conditions made as many errors as children in Baseline. Pairwise comparisons showed that children in Experiment 1 (*M*_1_ = 0.42 [0.24, 0.60]) made significantly fewer errors than children in Experiment 2 (*M*_2_ = 0.87 [0.69, 1.04]; *M*_1-2_ = -0.45, *p* = 0.002 [-0.76, -0.13]) and Experiment 3 (*M*_3_ = 1.22 [1.02, 1.40]; *M*_1-3_ = -0.80, *p* < 0.001 [-1.12, -0.47]). Moreover, children in Experiment 2 made fewer errors than children in Experiment 3 (*M*_2-3_ = -0.35 [-0.67, -0.03], *p* < 0.01, all comparisons are Bonferroni corrected).

The likeliest explanation for this seemingly paradoxical result is that in the present study, children made more errors because they were more faithfully generating the responses of the models in the order demonstrated than children in the 1 model demonstration condition, as was the case in Experiment 1. Because the model demonstrated opening the box before demonstrating the removal of the defenses, children in the demonstration conditions made a significantly high number of lift and slide errors, which were the responses they first observed the model make.

Given that there were no significant differences between 1 and 2 model demonstration conditions, we collapsed across demonstration conditions to compare individual error types between the three different experiments using a Kruskal–Wallis test. Results showed a significant difference in the number of slide and lift errors between experiments [Slide Error: χ^2^(2) = 24.72, *p* < 0.001, η^2^ = 0.11, Lift Error: χ^2^(2) = 34.60, *p* < 0.001, η^2^ = 0.14; Wrong Side: χ^2^(2) = 1.70, *p* = 0.43, η^2^ < 0.01; Destroy = χ^2^(2) = 1.62, *p* = 0.44, η^2^ < 0.01, Kruskal–Wallis test]. A post-host analysis using a Mann–Whitney test revealed that more children in Experiments 2 and 3 made slide (EXP_2-1_: *Z* = -3.20, *p* < 0.001, *r* = 0.26, EXP_3-1_: *Z* = 4.92, *p* < 0.001, *r* = 0.40) and lift errors (EXP_2-1_: *Z* = -4.76, *p* < 0.001, *r* = 0.38, EXP_3-1_: *Z* = -5.66, *p* < 0.001, *r* = 0.46) than children in Experiment 1. Children in Experiment 3 made significantly more slide errors (EXP_3-2_: *Z* = -2.31, *p* = 0.04, *r* = 0.19), but not more lift errors than children in Experiment 2 (EXP_3-2_: *Z* = -1.23, *p* = 0.52, *r* = 0.09, all analysis are two-tailed and Bonferroni adjusted).

### Discussion

Analysis of both target responses and errors in Experiment 3 are consistent with prior research showing that in the artifact domain, preschool age children are high-fidelity—overimitators—copying all demonstrated responses with little flexibility and regardless of their causal necessity or cost (Lyons et al., 2007, 2011; Nielsen et al., 2014a). Here, children in the 1 and 2 model demonstration conditions, after observing a model first opening the compartments and then removing the defenses (a violation of causality) followed suite, attempting to open the compartments as demonstrated, resulting in a high frequency of Slide and Lift Errors. These errors are notable as they were generally absent in the Baseline condition (c.f., **Figure 3**), serving as a proxy measure of social learning and imitation fidelity. And, as in Experiments 1 and 2, there was a non-significant trend for children in the 2 model demonstration condition to make more errors overall than children in the 1 model demonstration condition. Despite this high-frequency of errors, children in the 2 model demonstration condition, nonetheless, opened both compartments at rates greater than Baseline, evidence of summative imitation. The same was not true of children in the 1 model demonstration condition. While the 1 and 2 model demonstrations did not statistically differ, these results, nonetheless, suggest that children in the 2 model condition, generally, encoded and subsequently recalled the demonstrated events better than children in the 1 model condition.

## General Discussion

Overall, results showed that children in Experiments 1–3 showed robust evidence of summative imitation, imitatively combining different responses across different models to achieve a novel goal in a problem-solving task. Children in Experiment 2 succeeded in learning by summative imitation even when actions and goals were causally dislocated and presented by different models, making the function of responses opaque and the task more challenging. The flexibility of learning by summative imitation was further tested in Experiment 3. Results showed that children reproduced the demonstrated events (i.e., attempting to open compartments prior to removing defenses) as shown and failed to flexibly recombine the demonstrated events (i.e., remove defenses before opening compartments) prior to their first responses. As a result, children in Experiment 3 made significantly more errors than children in Experiment 1 (but not Experiment 2). However, after their first response, children evidenced more flexibility. For instance, following the first response, where children generally attempted to open a compartment without first removing the defense, children in the 2 model condition generated more target responses and successfully opened both compartments relative to children in Baseline. This result is consistent with a number of other studies showing that children are sensitive to their own mistakes in social learning tasks as well as the difficulty of the task (Williamson and Meltzoff, 2011; Wood et al., 2013). In one social learning study, children changed a previously rewarded response to a new alternative response demonstrated by a model (Wood et al., 2013). Children’s performance in the present study is consistent with these other studies and suggests that after making an error, children reconfigured, and perhaps restructured, the events they observed: removing the defenses prior to opening the compartments.

While there was some evidence that across experiments children in the 2 model condition learned better (albeit, often marginally so) than children in the 1 model demonstration condition, the underlying cognitive representations guiding responses in the 1 and 2 model condition do not appear to differ, given the similarity in children’s responses. An analysis of error patterns, for instance, showed no significant difference between 1 and 2 model demonstration conditions. Different representations underlying children’s performance in the 1 vs. 2 model conditions should have resulted in more robust and consistent differences in performance. Consider children’s performance in Experiment 3. Had children in the 1 model condition generated one continuous representation of the two action events, and children in the 2 model condition generated two independent representations of each action event that could be re-arranged flexibly, then children in the 2 model condition should have made fewer errors, than children in the 1 model condition. Yet, there were no significant differences in either the total number or the types of errors made by children in the two demonstration conditions.

There was also a tendency across Experiments for children in the 2 model condition to make more target responses relative to Baseline and imitate with higher fidelity (Experiment 1) than children in the 1 model demonstration condition. There are several possible explanations for this. First, the 2 model demonstration condition presented the same information as the 1 model demonstration condition in two discrete “chunks.” It has long been recognized in the cognitive sciences that grouping information into meaningful clusters has a facilitative effect on both encoding and recall (Miller, 1956; Terrace, 2001). While the present study was not designed to test such a possibility, it is nonetheless, possible that a type of ‘social chunking’ may explain the facilitative effect of learning different information from multiple models. However, besides improving encoding and recall, the present study offers no robust evidence that such chunking fundamentally altered how children in the 1 and 2 models demonstration conditions represented observed events. Second, as previously stated, observing multiple models has a facilitative effect on social learning (Bandura and Menlove, 1968; Schunk, 1987; Herrmann et al., 2013). One explanation for this facilitative effect may have to do with the fact that multiple models provide the child not just with more information but also with “normative” or culture-specific information which may add to the salience of the actions demonstrated (Keupp et al., 2013), increasing imitation fidelity (Herrmann et al., 2013).

Nonetheless, the unique temporal and spatial constraints associated with summative imitation might engage causal reasoning in a way that learning from a single model might not. As a result, certain summative imitation paradigms using different tasks and procedures might lead to distinct representations in the 1 vs. 2+ model demonstration conditions. As of yet, we do not know how (and whether) children combine different responses from models who are temporally as well as spatially separated.

The result that children tended to copy the specific (and causally ineffective) action sequence over the goal of the task, stands in contrast with results from another study showing that when executing different action sequences on different tasks, 3-years-old copy the goal structure of the sequences over the sequential structure of the demonstrated actions (Loucks and Meltzoff, 2013). Had children in Experiment 3, for example, encoded the goal structure rather than the specific sequence structure, they would have made few errors while opening the problem box. This discrepancy may be explained by the fact that in the present study models performed different actions sequences on different parts of the *same* apparatus, whereas in the Loucks and Meltzoff (2013) study a model demonstrated different action sequences on *different* tasks. Together, these results confirm that task type matters when learning by imitation (Subiaul et al., 2012, 2014). While children must regularly disambiguate multiple action sequences performed across different tasks (e.g., doing laundry and folding clothes), it is also the case that children must learn that the same object has multiple functional properties (e.g., the same tool may be used to hammer, cut or scrape). Both are critical aspects of cultural learning that may be represented differently in the brain. Understanding ‘why’ is a question that merits further exploration.

A possible limitation is that children observed the model reconfigure the box following each demonstration, proving children with additional causal information. However, the fact that children faithfully replicated the demonstrated technique even in Experiment 3 (i.e., attempting to open the compartments prior to removing the defenses) shows that children were not problem-solving by affordance learning, at least, not on the first trial. It is also an open question whether children are able to combine information if demonstrations are separated by long time intervals, as they might in a more natural setting. Results might also change if the demonstrations are separated spatially or presented across different mediums, such as video. While beyond the scope of the present study, answering these questions will shed light on the versatility and flexibility of children (and adults’) social and imitation learning skills as well as insight into the underlying cognitive systems mediating such learning.

The high-fidelity of children’s summative imitation indicates that learning and combining different types of information from multiple models may represent a more natural method or at least as natural and efficient a method as learning from a single model. It is certainly the case that in the physical domain, children are adept at synthesizing multiple pieces of information to make causal inferences (c.f., Gopnik and Schulz, 2004). The present study shows that children are equally adept at synthesizing different sources of *social* information in order to generate novel responses and solutions to complex problems. It is an open question whether the same causal processes used to synthesize information in the physical domain is responsible for piecing together different responses across models in the social domain, as some have suggested (Buchsbaum et al., 2012).

While the present study shows that children possess a mechanism that involves combining information across multiple models—summative imitation—it does not explain the range of information that can be learned and combined by summative imitation. The use of a problem box limited us to studying only problem-solving or innovation via combination (Lewis and Laland, 2012) and provided little room for novel innovation, as each possible manipulation of the box was demonstrated in all demonstration conditions. So, an important limitation of the present study is that results showed that children can solve a relatively simple problem by combining different responses by multiple models. However, we see this set of studies as a necessary first step for future research which should explore whether summative imitation may result in truly “novel” innovations involving more complex tasks or innovations that lead to better or more efficient solutions to problems (e.g., innovation via modification). But such limitations should not diminish the novelty and importance of these results, namely, that children despite more distractors (e.g., different models coming and going, delays between demonstrations), increasing the likelihood for errors, accurately imitated two distinct action events presented by two different models to solve a novel problem.

## Conclusion

While researchers disagree as to whether high-fidelity imitation is necessary for cumulative culture, there is a general consensus that cumulative culture requires both the creation (problem-solving/innovation) and social transfer (social learning) of others’ responses and knowledge (Tomasello et al., 2005; Boyd et al., 2011; Dean et al., 2012; Lewis and Laland, 2012; Legare and Nielsen, in press). But, to date, these research questions have been explored independently of one another, with research focusing on children’s ability to innovate or imitate in problem-solving tasks separately (e.g., Cutting et al., 2011, 2014; Beck et al., 2012). One reason for this being that while innovation has been conceptualized as an asocial—individual—learning process (Ramsey et al., 2007), imitation is thought of as the quintessential social learning mechanism (Over and Carpenter, 2012). This dissociation, however, has been challenged by meta-analyses showing that there is a strong association between social learning and problem-solving or innovation (Reader et al., 2011) and by computational models demonstrating that both high-fidelity imitation along with the combination of others’ actions (i.e., innovation by combination) best predicts cumulative culture (Lewis and Laland, 2012).

Here, we sought to empirically explore whether at least one type of problem-solving—innovation by combination (Lewis and Laland, 2012)—may be achieved by imitation. Results showed that preschool age children successfully opened a novel problem box by combining two different actions demonstrated by two different models, a process we refer to as summative imitation. Though previous studies have described young children as “cultural magnets” (Flynn, 2008), the psychological mechanisms supporting and furthering cultural evolution are very much in doubt (Caldwell and Millen, 2009; Call and Tennie, 2009; Heyes, 2012). Given the results reported here, we would like to further the hypothesis that the ease and fidelity with which young children combine information across models—summative imitation—may serve as a mechanism for cultural evolution by propagating and generating novel solutions to problems that in some contexts may lead to truly novel innovations.

## Conflict of Interest Statement

The authors declare that the research was conducted in the absence of any commercial or financial relationships that could be construed as a potential conflict of interest.

## Footnotes

1 It is important to emphasize that combining different imitative responses is not equivalent to emulation, which is characterized by selective imitation or replicating either the model’s intended goal or the achieved end-states using idiosyncratic means (Subiaul et al., 2015). In summative imitation, two or more demonstrated responses are faithfully imitated together.

2 Following Kummer and Goodall (1985) we will use problem-solving and innovation interchangeably throughout.
